# Recent Advancements and Perspectives in the Diagnosis of Skin Diseases Using Machine Learning and Deep Learning: A Review

**DOI:** 10.3390/diagnostics13233506

**Published:** 2023-11-22

**Authors:** Junpeng Zhang, Fan Zhong, Kaiqiao He, Mengqi Ji, Shuli Li, Chunying Li

**Affiliations:** 1 College of Electrical Engineering, Sichuan University, Chengdu 610017, China; junpeng.zhang@scu.edu.cn (J.Z.); zhongfan@stu.scu.edu.cn (F.Z.); mengqi12616@163.com (M.J.); 2 Department of Dermatology, Xijing Hospital, Fourth Military Medical University, Xi’an 710032, China; hekqiao@163.com

**Keywords:** dermatology, vitiligo, deep learning, machine learning, image segmentation, classification

## Abstract

Objective: Skin diseases constitute a widespread health concern, and the application of machine learning and deep learning algorithms has been instrumental in improving diagnostic accuracy and treatment effectiveness. This paper aims to provide a comprehensive review of the existing research on the utilization of machine learning and deep learning in the field of skin disease diagnosis, with a particular focus on recent widely used methods of deep learning. The present challenges and constraints were also analyzed and possible solutions were proposed. Methods: We collected comprehensive works from the literature, sourced from distinguished databases including IEEE, Springer, Web of Science, and PubMed, with a particular emphasis on the most recent 5-year advancements. From the extensive corpus of available research, twenty-nine articles relevant to the segmentation of dermatological images and forty-five articles about the classification of dermatological images were incorporated into this review. These articles were systematically categorized into two classes based on the computational algorithms utilized: traditional machine learning algorithms and deep learning algorithms. An in-depth comparative analysis was carried out, based on the employed methodologies and their corresponding outcomes. Conclusions: Present outcomes of research highlight the enhanced effectiveness of deep learning methods over traditional machine learning techniques in the field of dermatological diagnosis. Nevertheless, there remains significant scope for improvement, especially in improving the accuracy of algorithms. The challenges associated with the availability of diverse datasets, the generalizability of segmentation and classification models, and the interpretability of models also continue to be pressing issues. Moreover, the focus of future research should be appropriately shifted. A significant amount of existing research is primarily focused on melanoma, and consequently there is a need to broaden the field of pigmented dermatology research in the future. These insights not only emphasize the potential of deep learning in dermatological diagnosis but also highlight directions that should be focused on.

## 1. Introduction

**Figure 1:**
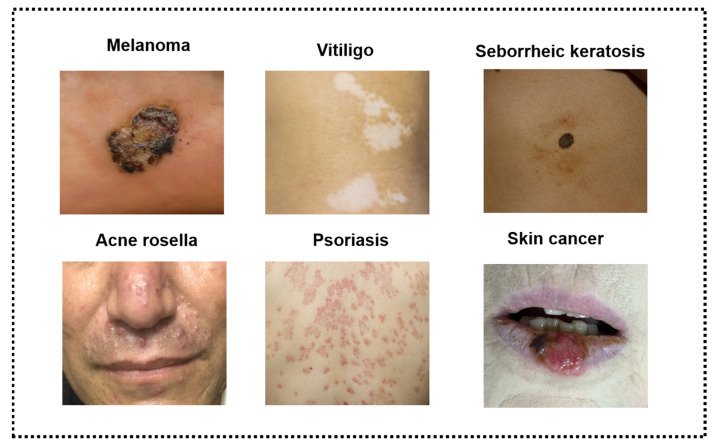
Chart of six common types of skin disease.

The skin is the largest organ in the body, and plays a crucial role in defending against environmental threats such as bacteria, viruses, and harmful substances. Skin diseases are a widespread health problem affecting people of all ages, and they can be caused by various factors such as genetics, lifestyle, and environmental exposure. Some of the most common skin diseases include acne, skin cancer, seborrheic keratosis, psoriasis, melanoma, and vitiligo, as depicted in Figure 1. Because of the progressive and prevalent nature of skin diseases, their adverse effects can significantly impact the physical and mental well-being of patients. Consequently, accurate and timely diagnosis is crucial for effective treatment and health management.

Recently, some skin diseases are still lagging behind regarding their clinical diagnosis and treatment. For example, there are some skin diseases that are difficult to diagnosis due to the lack of obvious pathological features in their early stages [1,2,3], such as skin cancer and vitiligo. Traditional diagnoses of dermatological diseases rely heavily on the visual inspection of skin features and subjective evaluations based on prior experience [4]. This approach often lacks precise, objective, and quantitative criteria; even dermatologists are not immune to the possibility of misdiagnosis. Even more unfortunately, in some remote areas where access to dermatologists is limited, non-dermatologists are often responsible for diagnosing and treating dermatological diseases with limited knowledge and training in this specialized field. Despite the availability of dermatology textbooks as reference material, these individuals still face significant challenges in achieving accurate diagnoses. The scarcity of dermatologists and an unbalanced distribution of healthcare resources exacerbate the difficulty of accurate diagnoses in such areas.

Fortunately, Artificial Intelligence (AI) technology based on image recognition has emerged as a promising approach for the diagnosis of skin diseases. AI algorithms can be trained with large datasets of skin images to learn the patterns associated with different skin diseases. This enables them to provide more accurate diagnoses than humans in some cases, particularly in cases where the disease is in its early stages. Additionally, through meticulous design and careful debugging, AI algorithms might be not subject to the biases of humans, which can lead to more objective diagnoses. As such, AI-based diagnostic tools have the potential to overcome some of the challenges associated with diagnosing skin diseases [5,6,7]. Currently, the most commonly used AI algorithms include machine learning (ML) and deep learning (DL) algorithms. Both of these can identify repetitive features of skin lesions and summarize them, allowing accurate diagnoses of benign and malignant lesions. DL typically exhibits superior performance when dealing with large datasets and complex features. However, ML methods can still be useful in certain situations, especially when the data size is limited. These approaches can be employed in computer-aided diagnosis (CAD) systems, offering precise classification outcomes for dermatologists. Additionally, for non-dermatologists, these systems can help to reduce errors resulting from limited expertise. Therefore, it is of great interest to delve into the evolution and recent achievements of ML and DL methods in the field of dermatological diagnosis, to identify the current challenges and offer appropriate recommendations to drive progress.

This paper provides insights into the application of ML and DL methods in dermatological diagnosis, assessing their achievements in the segmentation and classification of medical images, and conducting a review of existing issues. By comparing and summarizing these methods, the limitations of current research are elucidated, and future directions for development are proposed. It is expected that the improved algorithms can achieve more accurate diagnostic results and a faster speed of diagnosis to promote the further development of computer-aided diagnosis systems for skin diseases.

## 2. Materials

### 2.1. Study Selection

We searched four databases, including Pubmed, IEEE, SpringerLink, and Web of Science, for original English research papers. In the course of this analysis, only papers that had been published in journals and documented the appropriate scientific process were considered.

Papers were included based on the following inclusion criteria: (i) the presence of segmentation and classification algorithms for binary or multi-class skin lesions, (ii) the use of ML or DL methods, (iii) publication in English.

Exclusion criteria were used to exclude unrelated studies based on the following list of criteria: (i) review articles, (ii) case reports, (iii) books, and (iv) outdated literatures.

**Figure 2:**
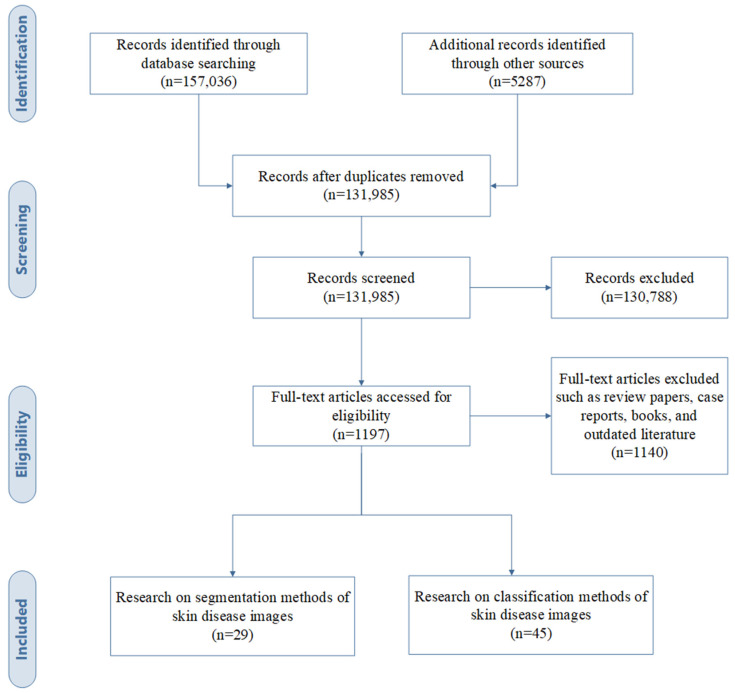
Flow diagram of the study selection using PRISMA.

The PRISMA flow chart in Figure 2 depicts the selection process [8]. The initial search yielded 157,036 eligible literature sources. These sources added to the 5287 records identified through other methods, such as forward and backward snowballing. After eliminating duplicate records, the number of papers was reduced to 131,985 records. Following the application of the inclusion criteria, we identified and further scrutinized 1197 full-text articles, applying specific exclusion criteria. The time frame for the research articles selected for this paper is 2015–2023, and progress made in the last five years was particularly emphasized. As a result, we selected 29 articles related to dermatological segmentation methods and 45 articles related to dermatological classification methods. The algorithms of the selected articles were divided into traditional ML methods and DL methods, and their results were discussed.

### 2.2. Datasets

In this section, the most commonly used datasets in this area of research are described. A wide range of available and free online datasets, such as DermNet, MED-NOOE, DermIS, ISIC 2017, ISIC 2018, ISIC 2019, ISIC 2020, and Derm7pt were used.

DermNet contains over 20,000 images of skin diseases, encompassing more than 200 different skin conditions and symptoms [9]. These images are acquired and evaluated by dermatological professionals; they categorize and label the images according to the type and severity of the skin condition.

**Figure 3:**
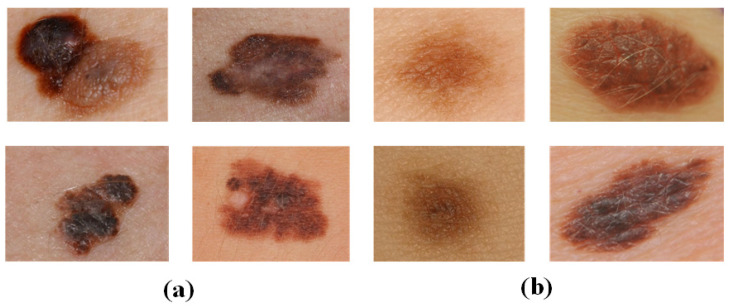
Different skin lesion examples in the MED-NODE dataset 10: (**a**) melanoma; (**b**) nevus.

The MED-NODE [10] dataset consists of 70 melanoma and 100 nevus images from the Digital Image Archive of the Department of Dermatology, University Medical Center Groningen (UMCG), which are used for the detection of skin cancer from macroscopic images. Figure 3 displays a selection of melanoma and nevus images.

DermIS is a large medical image dataset for the analysis and diagnosis of skin images [11]. This dataset, developed by the Medical Image Processing Laboratory at the University of Erlangen in Germany, includes more than 3000 images of dermatological diseases, covering more than 20 different types of skin conditions.

The ISIC 2017 dataset, provided by the International Skin Imaging Collaboration (ISIC) [12], includes 2770 high-quality dermoscopic images, including 1290 cases of benign skin, 1113 cases of malignant melanoma, and 367 cases of other types of malignant skin.

**Figure 4:**
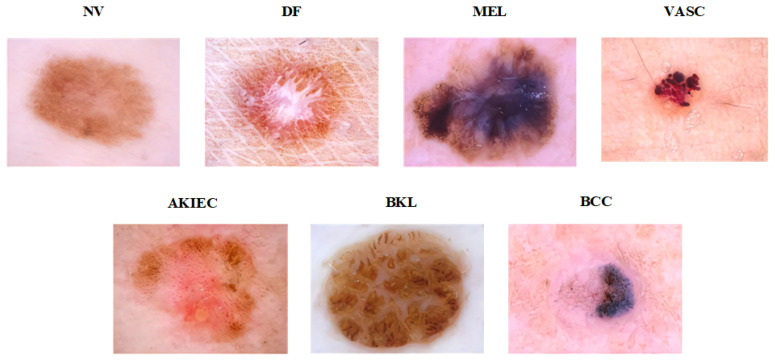
Different skin lesion examples in the ISIC 2018 [13] dataset.

The ISIC 2018 dataset is a publicly available dataset for skin image classification provided by the ISIC [13]. This dataset was compiled from data concerning all anatomical sites, except mucosa and nails, using various dermoscopy techniques from retrospective samples of patients who had been screened for skin cancer at multiple institutions. The training dataset consists of seven classes: actinic keratosis (AKIEC), basal cell carcinoma (BCC), benign keratosis (BKL), dermatofibroma (DF), melanocytic nevus (NV), melanoma (MEL), and vascular lesions (VASC). There are different numbers of images in each group. MEL has 1113, NV has 6705, BCC has 514, AKIEC has 327, BKL has 1099, DF has 115, and VASC has 142. Figure 4 presents the seven categories of skin disease images in ISIC 2018.

ISIC 2019 [14] consists of eight recognized classes and one outlier image class. These classes are MEL, NV, BCC, AKIEC, BKL, DF, VASC, and basal cell carcinoma (SCC). This dataset consists of 25,331 images, of which 867 are for AKIEC, 2624 are for BKL, 3323 are for BCC, 239 are for DF, 12,875 are for NV, 4522 are for MEL, and 628 are for SCC. The VASC has 253.

Another dataset from the ISIC is ISIC 2020 [15], containing 2000 dermoscopic training images of unique benign and malignant skin lesions from over 33,126 patients. Each image is associated with one of the individuals using a unique patient identifier. All malignant diagnoses have been confirmed via histopathology, and benign diagnoses have been confirmed via agreement among experts, longitudinal follow-up, or histopathology. The full publication describing all characteristics of this dataset is available in preprint form and has not yet been peer-reviewed.

Derm7pt (also known as Derm7k) is a dataset for the classification of skin disease [16] containing over 20,000 images of patients with skin diseases and their corresponding diagnostic labels. This dataset collects both clinical and nonclinical photographs, including real cases and synthetic data, to cover as many dermatological types as possible. The skin diseases in the Derm7pt dataset include common skin cancers, viral infections, fungal infections, allergic reactions, inflammatory and autoimmune diseases, etc.

### 2.3. Selection Criteria of AI Algorithms for Different Types of Skin Images

AI technology has found widespread applications in the field of dermatological diagnosis [17]. This is attributed to its capacity to address crucial challenges associated with dermatological diagnosis, including the diversification of skin conditions and the extraction of complex features. ML and computer vision techniques facilitate efficient and automated image classification, as well as rapid feature recognition [18].

When dealing with diverse sizes and types of dermatological images, the choice of modeling method becomes crucial. Common modeling approaches include Convolutional Neural Networks (CNNs) and Support Vector Machines (SVMs) [19]. The selection of the appropriate method closely depends on both the quantity and quality of the available samples. Given the different imaging modalities employed in clinical dermatology, such as dermoscopy, reflectance confocal microscopy (RCM), and very high-frequency skin ultrasound (VHF skin ultrasound), this choice becomes even more important. Table 1 summarizes the imaging criteria and applicable models for the three different imaging devices [20,21,22,23,24,25,26,27,28,29,30].

Deep neural networks are used to process clinical images and high-dimensional representations of the local skin features are extracted. The disease status of skin regions in photographs can be explained via the skin surface’s properties, represented in the images [31]. In order to obtain the decision result of the diagnosis, the pictures are analyzed and the retained characteristics are entered into the classifier [32,33,34].

## 3. Methods

This section offers a comprehensive overview of DL and ML algorithms tailored for the field of dermatology. These algorithms are categorized into two distinct but interrelated domains: segmentation and classification of skin diseases. By examining these two critical areas, we delve into the advanced techniques and methods that play a vital role in enhancing the diagnosis and analysis of skin conditions.

### 3.1. Segmentation Methods

**Figure 5:**
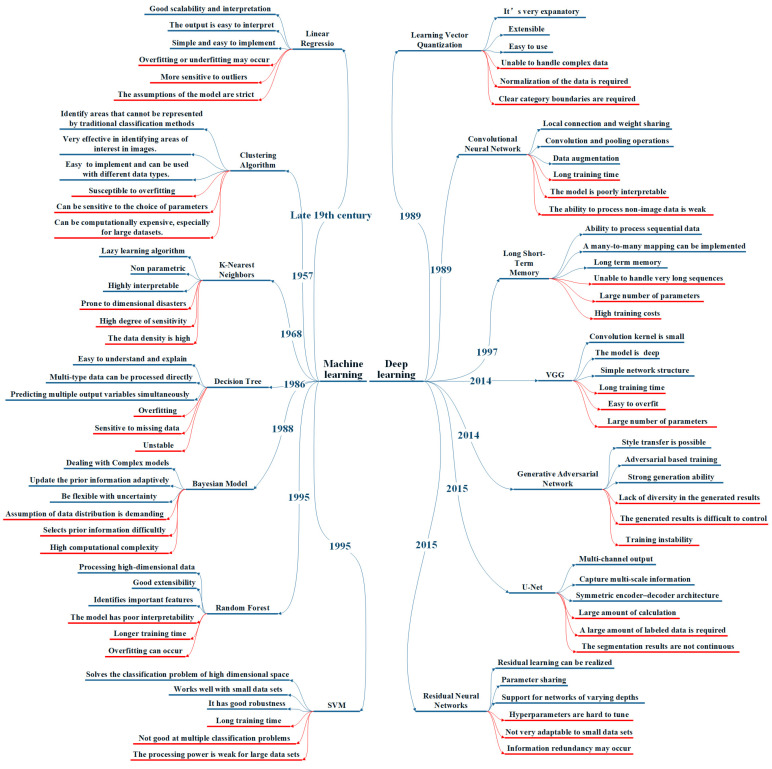
Tree-structured illustration of the advantages and disadvantages of traditional ML and DL models in image processing (red lines indicate disadvantages, blue lines indicate advantages) [36,37,38,39,40,41,42,43,44,45,46,47,48,49,50].

In the domain of medical imagery, traditional recognition of images mainly relies on edge segmentation and feature extraction methods [35]. For all skin diseases, the accurate segmentation of skin images is essential for detecting and localizing lesions. In this section, we explore two distinct approaches, encompassing DL-based methods and traditional ML processing techniques, with a particular focus on the emerging research trend concerning DL-based methods. Figure 5 summarizes the advantages and drawbacks of both traditional ML and DL models in the context of image segmentation and classification applications [36,37,38,39,40,41,42,43,44,45,46,47,48,49,50].

#### 3.1.1. Traditional Machine Learning

Numerous techniques are employed for the segmentation and feature extraction of skin disease images. In 2015, a segmentation method was proposed for vitiligo lesions on the posterior trunk using Linear Spectral Clustering (LSC) superpixels and Random Forests [51], resulting in improved segmentation outcomes. Unfortunately, this method is only suitable for smooth, normative skin and not for other types of skin, such as skin with lesions. Alam et al. [52] proposed a color-based segmentation method using K-means clustering, complemented with morphological image processing techniques. This method can accurately segment eczema lesion areas with a classification accuracy of 90%. However, its dependence on color information makes it unsuitable for other skin lesion types. Building upon [52], Thanh et al. [53] proposed two adaptive approaches, utilizing the normalization of color models, namely RGB and XYZ, to estimate global thresholds of skin lesion segmentation. Their method outperforms the Otsu segmentation method in terms of grayscale model segmentation. Yet, similar to [52], it exhibits limited adaptability for specific lesions and skin colors. Chica et al. [54] and Nurhudatiana et al. [55] introduced Independent Component Analysis (ICA) and Fuzzy C-Means (FCM) for vitiligo segmentation, respectively. In the ICA method, melanin images derived from ICA were processed using area growth techniques to segment vitiligo lesions. Both methods are only applicable to lesions on smooth skin and overlook facial lesions, with a relatively small sample size. Dash M et al. [56] introduced an image segmentation system grounded in the Bayesian model. This approach extracted 859 features and integrated swarm intelligence algorithms with traditional K-means and FCM clustering algorithms. This method exploits multiple features and clustering algorithms to improve the segmentation accuracy, but also has the drawback of increasing the computational complexity.

The above segmentation methods are all traditional ML methods, which are heavily dependent on threshold settings for image analysis. If the threshold value is not set reasonably, it will decrease the generalization ability of the model and lead to false recognition. Therefore, in terms of image segmentation, DL holds several advantages over traditional ML methods:

(1) Enhanced feature learning: DL models possess robust feature learning capabilities, automatically deriving advanced feature representation from data, in contrast to the manually designed features relied upon by ML models.
(2) Improved generalization: DL models exhibit strong generalization capabilities, performing well with small datasets, whereas ML models typically demand extensive manual feature engineering and large datasets.
(3) Scalability: DL models can be efficiently trained on large-scale data and manage such datasets rapidly through distributed computing, whereas ML models struggle due to their need for manual feature design.
(4) Flexibility: DL models offer a more adaptable structure, accommodating a wide range of tasks and data types, unlike ML models, which require manual design for distinct tasks and data types.

In conclusion, within the aspects of image segmentation, DL excels over ML due to its superior feature learning capabilities, generalization scalability, and flexibility, making it a valuable asset in practical applications.

#### 3.1.2. Deep Learning

**Figure 6:**
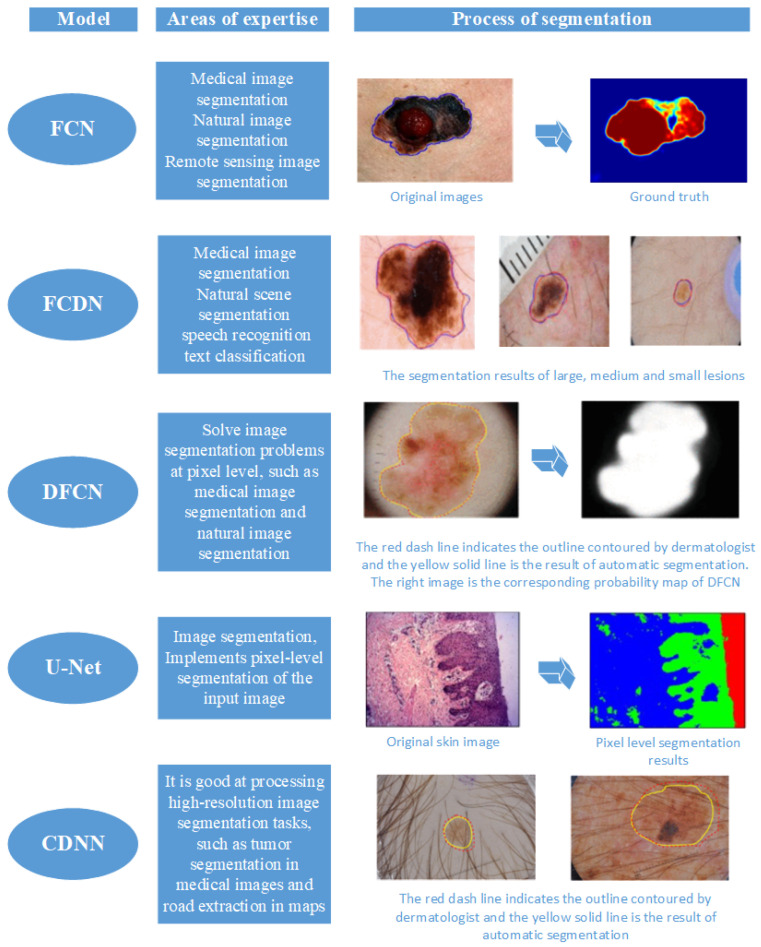
Five kinds of image segmentation models and their fields of expertise [57,60,61,62,63].

With the development of DL, some architectures have shown great potential in image segmentation tasks, such as U-Net, Fully Convolutional Networks (FCNs), and Fully Connected Neural Networks (FCNNs). The application of DL in medical image segmentation has become a popular topic of research [57,58,59,60]. Several CNN-based segmentation methods have been devised, such as the Fully Convolutional Deconvolution Network (FCDN) [61], Deep Fully Convolutional Network (DFCN), founded on the Jaccard distance method [62], Convolutional–Deconvolutional Neural Network (CDNN) [63], U-shaped FCN, and multi-stage FCN [64]. All of these methods are based on the integration and development of FCNs, demonstrating their superior segmentation accuracy. Nevertheless, they often need substantial datasets for effective training and may exhibit limitations in capturing segmentation details and low-intensity regions. Figure 6 provides an overview of five image segmentation models and their respective domains of expertise.

Some U-Net-based CNN models have been used in the study of skin lesion segmentation studies. These approaches typically demand fewer resources and smaller training datasets. Tschandl et al. [65] designed an FCNN with a U-Net style architecture, incorporating a pre-trained ResNet34 as the encoding layer for lesion segmentation, which is a preliminary step in the diagnosis of skin cancer from dermoscopic images. This method may be helpful when only a limited number of labeled samples are available for segmentation training. Unfortunately, since it is based on ImageNet pre-training, this method requires an adequate amount of segmentation training data in order to work. Peng et al. [66] reformulated the U-Net model for melanoma lesion detection based on [65], and proposed an adversarial network to address overfitting issues stemming from simpler samples. Adversarial networks can enhance the quality of segmentation results by generating adversarial data. While this approach helps to improve accuracy and robustness, it consumes significant computational resources and time in training and optimizing the adversarial network. Additionally, the choice of network architecture and hyperparameters may impact the segmentation outcomes. Adegun et al. [67] introduced a skin lesion segmentation method employing an FCN based on the U-Net architecture, enhancing the model through batch normalization. FCNs can efficiently process spatial information in the images and generate pixel-level segmentation outcomes, showing high accuracy and robustness in segmentation tasks. Thanh D N H et al. proposed a method that combines VGG-16 coding with U-Net architecture for skin lesion segmentation [68]. Their approach optimally utilizes the U-Net’s strengths, particularly in the case of limited training data. These studies emphasize the potential of U-Net-based Convolutional Neural Networks (CNNs) in skin lesion segmentation. Compared to traditional methods, the U-Net architecture exhibits the advantage of accommodating smaller training sets and adeptly mitigating overfitting. Furthermore, its performance can be further elevated through the addition of supplementary layers.

In addition to U-Net, there are other CNN-based architectures that have been used in this field. The Multi-Scale Fully Convolutional DenseNets (MSFCND) model [69] is a CNN-based approach capable of segmenting images of arbitrary sizes after training. Compared to U-Net, one of the most commonly used FCNs in medical image analysis, this method excels in most evaluation metrics. This superiority can be attributed to its integration of multiscale contextual information in the downsampling paths and the incorporation of multiscale depth supervision in the upsampling paths. Nasr-Esfahani et al. [70] introduced the Dense Pooling Full Convolutional Network (DPFCN) model, designed to segment damaged regions in skin images using dense pooling layers. This network overcomes limitations observed in many contemporary state-of-the-art segmentation methods, especially in the boundary detection phase, and outperforms other algorithms in skin lesion segmentation. In [71], an enhanced skin lesion segmentation model, based on deformable three-dimensional convolution and ResU-next++ (D3Dc-resu-next++), was proposed. Traditional neural network models often struggle to extract deep image features and are susceptible to gradient vanishing during backpropagation. In contrast, the 3D convolutional structure in this model effectively extracts diverse levels of image information, enhancing its efficiency. The use of ResU-NeXt++ stitches together different information levels obtained via the 3D convolutional structure, while the improved dynamic convolutional layer can better prevent the gradient disappearing, which significantly enhances the CNN’s ability to model geometric transformation. At the same time, the transfer learning method applied to D3Dc-ResU-NeXt++ emphasizes the contrast between the lesion area and normal areas, thus improving segmentation efficiency and robustness, and the adoption of RAdam further accelerates the convergence rate of the neural network. In [72], a method combining U-Net with an empty spatial pyramid pool (ASPP) is presented for the segmentation of skin lesions. This CNN model is an enhanced U-Net architecture, which includes an ASPP module to improve the effectiveness. Additionally, extended convolution allows the kernel to capture larger perceptual regions for better feature learning, with the incorporation of a batch normalization layer to normalize the feature map. This method primarily targets mobile platform applications with lightweight models. Although it achieved impressive performance with the ISIC challenge dataset, further validation of its generalization ability is warranted.

In 2023, Yuta et al. [73] developed a CNN-based skin lesion region segmentation system using the DeepLabv3+ model. Their CNN-generated segmented images were closely similar to those manually cropped by dermatologists. To enhance the accuracy of skin lesion detection, this research team adopted a strategy of segmenting multiple regions from the original images, enabling the extraction of attributes from various skin lesions. This approach significantly improved the segmentation accuracy of the CNN model, facilitating a clearer distinction between lesion areas and normal skin regions (e.g., around the lips and eyes). The application of this skin segmentation system resulted in an impressive 90% detection rate for infectious diseases. Nevertheless, their study faces limitations, notably the relatively small sample size and significant data variation among different types of skin lesions, potentially introducing bias into the DL process. To enhance the robustness of CNN-based skin lesion segmentation systems, the need for more extensive image data in training is evident. It is worth noting that a recent prospective study highlighted the superior performance of AI over general practitioners, particularly when working with balanced datasets [74]. In the same year, a state-of-the-art approach for segmentation was presented by Dasari et al. [75]. This method utilized the newly developed W-EFO algorithm combined with enhanced U-Net to improve the segmentation of skin damage characteristics by analyzing the fitness function. Their proposed method sources input images from three distinct datasets and preprocesses them through grayscale conversion, color removal, and contrast enhancement. Through experimental analysis, the provided W-EFO-E-CNN with enhanced U-Net achieved a remarkable 98% accuracy across all datasets. This skin lesion model surpasses conventional methods in terms of various optimization techniques, segmentation methods, and classifiers. However, it is important to note that further inclusion of clinical data is necessary to mitigate potential errors in the data samples.

**Figure 7:**
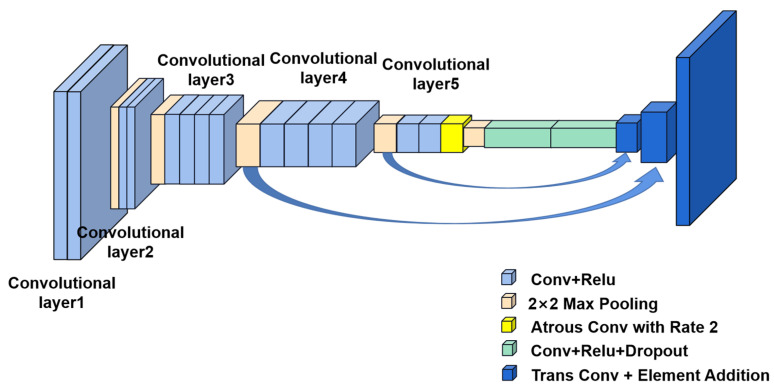
Network structure of an FCN with a U-Net structure and transposed convolutional layers. The lowest convolutional layer is combined with RelU activation functions. After multi-layer convolution, the dropout function is used for optimization [79].

In the domain of vitiligo image segmentation, a weakly supervised approach was introduced by Bian et al. [76]. This method requires only class labels of clinical images, employing a deep CNN to generate activation maps and produce image segmentation results. The effectiveness of this technique is highlighted by its unique ability to integrate activation maps with hyperpixel-based saliency propagation, resulting in segmentations that preserve the edges of lesions exceptionally well. Rigorous evaluations have confirmed the superiority of this method, representing the pioneering use of DL algorithms in the field of vitiligo image segmentation. Furthermore, the development of the Vit2019 dataset, comprising 2000 vitiligo images, opens doors for the use of data-driven algorithms in subsequent studies. The accurate segmentation of lesion skin borders is crucial for measuring the progression and severity of vitiligo. Nevertheless, many existing methods still rely on human intervention, even within weakly supervised settings. Low et al. [77] introduced a U-Net-based CNN with great potential for vitiligo segmentation. CNNs, acting as enhanced feature extractors, use U-Net with modified shrinkage paths to generate the initial lesion segmentation. High-confidence pixels are subsequently utilized as “seeds” for segmentation through the watershed algorithm, leading to rapid and reliable segmentation without manual intervention. This method notably reduces the time required for manual segmentation, thereby optimizing computer resource utilization and reducing the necessity for extensive post-prediction corrections. It is crucial to note that this study is limited by a scarcity of data resources and would benefit from further validation with more extensive datasets. Khatibi T et al. [78] presented a method for locating vitiligo lesions in skin images using the Stack Ensemble of Deep and Conventional Image Segmentation (SEDCIS) method for unsupervised stack integration. This localization of vitiligo lesions facilitates precise segmentation and evaluation of their surfaces. While this method demonstrated a notable Jaccard score of 0.94, it is primarily suited for segmenting patchy vitiligo and may not be optimal for full-face vitiligo segmentation. In 2021, an advanced automatic segmentation method for facial vitiligo was presented [79], breaking away from the limitations of previous methods confined to normative skin or vitiligo patches. This algorithm, employing an FCN with a U-Net structure and transposed convolutional layers (as illustrated in Figure 7), achieved superior segmentation results. U-Net, widely adopted for biomedical image segmentation, excelled in generating precise segmentation boundaries due to its symmetric shrinkage and expansion paths. Moreover, transposed convolutional layers enhanced the upsampling performance. Notably, during the convolution process, differential weighting was applied to vitiligo and non-vitiligo pixels to emphasize the former during training. The algorithm’s results outperformed all existing automatic vitiligo segmentation methods and even surpassed the visual assessments of the two dermatologists involved in the study.

### 3.2. Classification Methods

This section provides an overview of the ML and DL approaches used in the classification of skin diseases. We particularly emphasize the application and in-depth analysis of DL methods. By exploring these techniques, we aim to provide a comprehensive understanding of advances in the field of dermatological diagnosis and the role of DL within them.

#### 3.2.1. Traditional Machine Learning

The ultimate objective of CAD systems is the classification of medical images. Before the advent of DL, the standard practice involved the manual definition of image features, including texture, image shape, and grayscale histograms, among others. Subsequent to feature selection, ML models, such as SVMs, logistic regression, and Random Forests, were widely employed for classification. Notably, in the domain of dermatological classification, SVM, K-Nearest Neighbor (KNN), and Naive Bayes models have been prevalent [80,81,82,83,84,85].

In a study conducted by Pennisi et al. [86], the performance of four classifiers—Adaboost, Naive Bayes, KNN, and Random Forest—was evaluated for distinguishing between benign (common and atypical nevi) and malignant lesions (melanoma). Adaboost emerged as the optimal performer among these classifiers. However, the study found that while the segmentation method exhibited very high accuracy in handling benign lesions, its accuracy significantly decreased when dealing with malignant lesions. Consequently, this algorithm’s results may not be sufficiently accurate for malignant lesions. In 2015, a novel approach for the automatic classification of skin diseases was introduced, combining KNN and an SVM for dermatological disease classification [87]. The individual accuracy of KNN-based classification was 76%, and SVM-based classification achieved 78%, while the fusion of these two classifiers reached an accuracy of approximately 85%. This innovative approach represented a pioneering effort in classifier fusion, demonstrating the superior performance of the combined classifier. Nevertheless, the system’s overall performance was hindered, primarily due to degradation in certain classes resulting from the heterogeneity in data resources. In 2016, Suganya et al. employed K-means clustering to segment skin lesions in dermoscopic images, using an SVM classifier for the classification of skin diseases, particularly melanoma [88]. While most classification methods have primarily concentrated on melanocytic skin lesions, this approach proved effective for high-precision classification of skin cancers in both melanocytic skin lesions and non-melanocytic skin lesions within the epidermal layer. Future research should aim to address the segmentation of skin lesions in the dermal layer. In 2017, Rahman et al. proposed a similar method using KNN and SVM classifiers [89]. Their approach incorporated color thresholding for image segmentation, followed by higher-order statistical analysis of various color planes. Extracted features were subjected to SVM and KNN classifiers for robust classification. The integration of rigorous preprocessing and feature extraction from standard digital images led to an improvement in classification accuracy.

In 2018, a multi-classification CAD system that combines more than two classifiers was developed, employing five classifiers: decision tree, KNN, SVM, and fusion classifiers with diverse kernels in conjunction with Artificial Neural Networks for dermatological classification. The study concluded that the SVM classifier, featuring non-linearity, demonstrated the best performance [90]. While the classification results for skin lesions were not exceptional, this research accomplished the classification of six diseases and offered cost-effective dermatological care through image processing and ML algorithms. In 2019, Murugan et al. [91] proposed a watershed segmentation method to classify skin images, enhancing the segmentation accuracy and consequently the classification accuracy. Their method integrated extracted features, including shape characteristics, the ABCD rule, and GLCM. Notably, the SVM’s implementation of the ABCD rule exhibited a progressive evaluation phase using an innovative classifier technique. In comparison to Random Forest and KNN, the SVM demonstrated superior results, characterized by high accuracy and robustness, along with commendable out-of-sample generalization. In 2020, Balaji et al. introduced a dynamic graph-cutting algorithm for skin lesion segmentation and applied a plain Bayesian classifier for classification [92]. The classifier demonstrated ease of implementation, rapid output prediction, and commendable performance, even in multi-class prediction scenarios. It is essential to acknowledge that the classification methods mentioned above rely heavily on feature extraction from segmented lesion images, leading to the performance being contingent on the quality of feature selection. In contrast, DL methods have shown superior capability in addressing this challenge.

#### 3.2.2. Deep Learning

**Figure 8:**
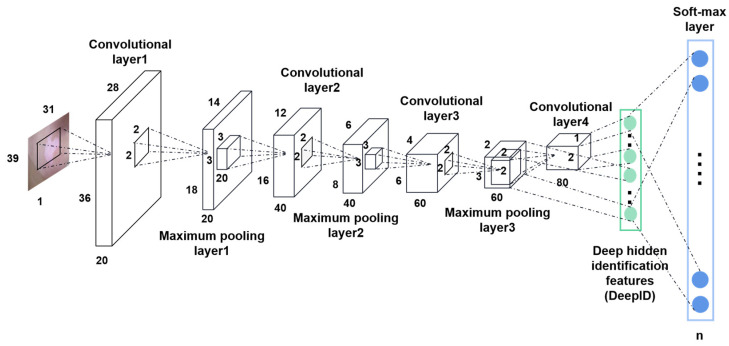
Classification of skin diseases using a CNN [93]. This network contains four convolutional layers (three maximum pooling layers) for hierarchical feature extraction, followed by a fully connected DeepID layer and a Soft-max output layer for classification. This model takes 39 × 31 × 1 inputs and predicts disease categories (n). When the input size changes, the height and width of the graph in the subsequent layer changes accordingly.

DL, as a subset of ML, exhibits considerable promise compared to traditional ML algorithms. In the domain of medical image classification, with the rapid advancement of DL models, particularly the widespread adoption of deep CNNs, establishing neural network models has been regarded as a prevailing approach for automatic feature extraction and dermatological classification (as depicted in Figure 8, illustrating the process of classifying skin diseases using DL). Notably, Nasiri et al. [93] introduced a classification approach for skin lesions founded on CNNs, selecting a CNN with 16 convolutional and pooling layers for iterative training and learning. This method significantly enhanced the image classification efficiency and underwent rigorous validation with 1796 dermoscopic images. Nevertheless, its applicability is currently limited to melanoma classification. Ahmad et al. [94] presented a hybrid classification method that combines a deep Convolutional Neural Network (DCNN) with stacked Bidirectional Long Short-Term Memory (BLSTM) networks. Their proposed model capitalizes on the strengths of both approaches, mitigating classification inaccuracies and irrelevant feature selection, especially in scenarios involving highly similar images of malignant and benign lesions. This process entails the extraction of deep features from facial images through the CNN, followed by the input of sequential features into the dual BLSTM network, ultimately leading to dermatological image classification through the Soft-max function. Through extensive testing on two dermatological datasets to enhance generalizability, their method achieved an impressive average accuracy of 91.73%. In contrast to contemporary dermatological classification methods, this approach represents a significant advancement in dermatology classification. With the availability of powerful computational resources, more complex DL systems are under development. Nonetheless, sometimes these complex systems take too much time to train and are therefore inefficient. In 2022, an Eff2Net model was introduced [95], which incorporates the Efficient Channel Attention (ECA) module to replace the standard Squeeze and Excitation (SE) module within EfficientNetV2. This deliberate modification effectively decreases both the number of trainable parameters and those learned by the CNN. The EfficientNet model is well-known for its ability to achieve high accuracy within a shorter time and fewer parameters. The ultimate results demonstrate that this model achieved superior classification performance with a lower computational complexity.

In a study conducted by Mohammed et al. [96], a dermatological classification algorithm was proposed that employs a combination of SVMs and Back Propagation Neural Networks (BPNNs). Their methodology commenced with an adjustment of elements through the application of regularized Random Forests, followed by the implementation of image enhancement techniques. The outcome of this research was remarkable, with an impressive classification accuracy of 99.7% and sensitivity of 99.4%. However, it is essential to note that the dataset they considered comprises only 400 images, underscoring the need for further validation using a more extensive dataset to robustly establish this method’s validity. Zia Ur Rehman et al. [97] sought to enhance the performance of pre-trained MobileNetV2 and DenseNet201 models through the addition of supplementary convolutional layers, aiming to optimize skin cancer detection. This method specifically targeted the discrimination between benign and malignant categories of skin cancer, leading to a notable accuracy rate of 95.50%. Additionally, this study introduced Gradient-Weighted Class Activation Mapping (Grad-CAM) visualization, representing a pioneering step toward an interpretable classification algorithm in the field. In the context of dermatological studies, CNNs often rely on classical loss functions, which restricts their ability to learn discriminative features from skin images.

To overcome this limitation, Ahmad et al. [98] presented an innovative approach utilizing the ResNet152 and InceptionResNet-V2 models, combined with a triplet state loss function. This pioneering research was the first to employ the triple loss function in dermatological images. Their approach involved embedding input images into Euclidean space using the deep CNN ResNet152 and InceptionResNet-V2 models, calculating L-2 distances in the Euclidean space through the triple state loss function to extract discriminative features of dermatological images. This approach had improved accuracy compared to many existing approaches for such dermatological tasks. Wu et al. [99] introduced a classification methodology that utilizes transfer learning-based CNNs for the classification of facial skin. Their approach employed five prominent CNN architectures, which were initially pre-trained on extensive datasets. These architectures include ResNet-50, Inception-v3, DenseNet121, Xception, and Inception-ResNet-v2. Transfer learning was skillfully applied to these models, and through a meticulous performance comparison, it was evident that the model demonstrated commendable performance across the six facial skin classification tasks. Furthermore, their models consistently achieved higher average accuracies when utilizing transfer learning, reaffirming the efficacy of this approach in the domain of dermatological image classification.

In a separate contribution, Hosny et al. [100] proposed a DCNN methodology tailored for the precise classification of melanoma. Notably, this approach adeptly addressed the complexities arising from variations in skin texture. To counterbalance the data deficiencies in existing DL-based methods, their methodology comprised a multi-step process. It commenced with preprocessing the input image, followed by the segmentation of regions of interest (ROIs). Then, they employed rotations and shifts to enhance the segmented ROI image. The performance of this novel approach was extensively evaluated using various DCNN architectures, including Alex-net, ResNet101, and GoogleNet, across multiple datasets, including MED-NODE, DermIS and DermQuest, and ISIC 2017. The results distinctly indicated that their approach achieved optimal classification, particularly when employing the enhanced GoogleNet architecture. Simultaneously, the research team introduced an additional high-precision method designed for a seven-class classification task [101]. This approach utilized the pre-trained AlexNet as a foundational framework. While the parameters of the original model served as initial values, the weights of the three replacement layers were randomly initialized. Rigorous testing was conducted using ISIC2018, resulting in notable success. The proposed method effectively classified skin lesions into seven distinct categories, demonstrating impressive performance in metrics including accuracy, sensitivity, specificity, and precision, with values reaching 98.70%, 95.60%, 99.27%, and 95.06%, respectively. Notably, this achievement surpassed the outcomes of existing studies by a margin of at least 6%. Subsequently, the team progressed their work to formulate a model capable of effectively handling an eight-class classification task [102]. They harnessed the power of transfer learning by employing a pre-trained model from GoogleNet, initializing parameters and further refining them through training. However, meticulous testing unveiled a certain level of randomness in their classification results, attributed to an imbalance in the distribution of sample images within the dataset. In a separate study [103], a groundbreaking approach was introduced, featuring a novel Residual Deep Convolutional Neural Network (RDCNN) tailored for diagnosing skin cancer lesions. This innovative model, trained and tested across six distinct skin cancer datasets, demonstrated its proficiency in extracting crucial features with exceptional precision, even when dealing with datasets of limited size. Worth noting is the inclusion of innovative shortcut connections in the RDCNN, setting it apart from established architectures such as ResNet. The performance of their proposed RDCNN classification model consistently outperformed existing methods for skin lesion classification. However, it is important to highlight that this model excelled in binary classification tasks, leaving room for further improvement in its performance for multi-class scenarios.

In a recent investigation [104], a CNN based on the InSiNet architecture was introduced to distinguish benign and malignant skin cancer lesions. This study involved testing the model under consistent conditions, utilizing 10,000 images from several ISIC datasets. Remarkably, the proposed algorithm achieved a classification accuracy of 94.59% with the ISIC 2018 dataset. This achievement prompted a comparative assessment of the proposed algorithm against seven other ML technologies, including GoogleNet, DenseNet-201, ResNet152V2, EfficientNetB0, RBF SVM, logistic regression, and Random Forest [105,106,107]. The distinctive strength of this approach lies in the InSiNet architecture, characterized by its reduced parameter count, lightweight models, shorter processing times, and superior accuracy. This efficiency is, in part, attributed to the selective discarding of irrelevant data that often constitutes noise in skin cancer lesion analysis. The elimination of such noise contributes positively to algorithm performance. Consequently, to further elevate classification accuracy, efforts should focus on enhancing the segmentation process.

In the year 2023, a hybrid CNN architecture that amalgamates DenseNet and residual networks was proposed for skin lesion classification tasks [108]. This innovative approach simplifies the intricate task of classifying skin lesions by training a unified CNN architecture to handle multiple classification challenges. Comprehensive analyses of the study results illustrate that superior performance can be obtained when multiple DL algorithms are combined. This methodology effectively decreases the complexity associated with the features present in skin lesion images and mitigates the need for the extensive parameter tuning required by conventional CNN models. Subsequently, Vatsala et al. introduced a fusion model that capitalizes the strengths of U-Net and CNN models [109]. Initially, U-Net was employed to localize and extract ROIs from dermoscopic images, followed by CNN-based multi-class classification on these segmented images. The optimization of the model’s performance was facilitated by employing both Adam and Adadelta optimizers. The experimental findings conclusively demonstrate that this fusion model surpasses other state-of-the-art techniques across all performance parameters. Furthermore, Paravatham et al. [110] proposed a DCNN model that incorporated global average pooling and preprocessing techniques. This approach aims to mitigate the common issue of overfitting in conventional deep CNN models and enhance the early-stage accuracy of skin cancer detection. In addition, they thoughtfully designed a user interface for evaluating the effectiveness of this DCNN model. It is important to highlight that this study, like its predecessors [108,109], employed the HAM10000 dataset, which is afflicted with data class imbalance. This imbalance problem often leads to the model being biased toward predicting the majority of categories while neglecting the minority. Consequently, while the overall accuracy may be high, classification accuracy for specific categories can significantly suffer. In a recent pioneering work, Gan et al. [111] introduced a cutting-edge multimodal transformer algorithm. This innovative approach integrates two encoders for processing images and metadata, along with a decoder for fusing multimodal information. The dataset used in this study comprised dermatological images and clinical metadata. Within the network, a Visual Transformer (ViT) model adept at deep feature extraction from images was skillfully employed. Metadata, conversely, were treated as labels and embedded using a novel soft label encoder (SLE). The decoder section introduced a novel mutual attention (MA) module designed to optimize the fusion of image and metadata features. In comparison to preceding state-of-the-art methods, this model exhibited superior performance, representing a significant advancement in the field of dermatological diagnosis.

While these DL methods have demonstrated impressive results and are extensively adopted for classifying neoplastic and inflammatory skin diseases, their application in the intelligent diagnosis of pigmented skin diseases, specifically vitiligo, remains notably limited. This limitation arises from challenges encompassing inadequate data volumes, a lack of specific targeting for vitiligo, and diminished detection accuracy. Further research is imperative to address these limitations and broaden the scope of AI applications in the diagnosis of pigmented skin diseases.

In 2019, a method employing CNNs for intelligent vitiligo detection was introduced [112]. This method underwent training on three distinct CNN models, namely Resnet50, VGG16, and Inception v2. Importantly, the training incorporated three different color space representations (RGB, HSV, YCrCb) on the same vitiligo dataset, with evaluation metrics encompassing accuracy, sensitivity, and specificity. As vitiligo lesions exhibit distinct contrast features under varying color space transformations, these three models fully leverage complementary color information, leading to substantial performance enhancements. This innovative approach exemplifies the pioneering strides made using DL within the domain of vitiligo diagnosis. In 2020, a vitiligo diagnosis system was introduced, comprising three essential components [113]. A distinguishing feature of this system is the utilization of images generated through a Cycle Consistent Adversarial Network (Cycle GAN) under a wood lamp. Furthermore, this system incorporated an advanced super-resolution technique, Attention-Aware DenseNet with Residual Deconvolution (ADRD), to enhance image resolution. Finally, the classification aspects of the system relied on Resnet50. Through comparative analysis with images lacking preprocessing, a substantial improvement in classification accuracy was observed, affirming the effectiveness of the applied preprocessing method. In 2022, Guo et al. proposed a hybrid DL model for vitiligo lesion detection [114]. This hybrid model integrates three diverse datasets and employs three distinct models. Their approach utilizes YOLO v3 for lesion localization, and UNet++ for lesion segmentation, and combines the outputs of these models for comprehensive testing. A notable advantage of this hybrid model is its proficiency in detecting small lesions, a valuable feature considering the variability in the number and size of vitiligo lesions. It is essential to highlight that, currently, this model is primarily suited for evaluating the severity of vitiligo lesions in individuals of Asian ethnicities. Further assessments are imperative to determine its applicability to other ethnic skin types. An alternative approach for vitiligo detection involves the utilization of the Learning Vector Quantization (LVQ) neural network [115]. LVQ is an artificial neural algorithm based on supervised learning that is trained using known data. The algorithm categorizes images into affected and unaffected areas, employing learning vectors for quantitative vitiligo assessment. This method achieved an impressive classification accuracy of 92.22%.

Collectively, these methods highlight the widespread application of DL techniques in the diagnosis of skin diseases, demonstrating remarkable effectiveness. As algorithms continue to enhance and data quality continues to improve, the accuracy of CNN models in classifying common skin diseases is steadily rising. Among the various neural network types, CNNs stand out for their ability to handle complex samples.

The DL methods for dermatological classification discussed in this section predominantly center around CNN-based methods and customized hybrid CNN networks. Consequently, it becomes evident that CNNs occupy a pivotal role and hold substantial significance in the field of dermatological classification [116,117,118,119,120,121,122].

## 4. Results

### 4.1. Indicators of Evaluation

In dermatological research, assessments of ML and DL models are rigorously conducted using a suit of standardized metrics. These metrics include accuracy (AC), sensitivity (SE), specificity (SP), the Dice score (DI), and the Jaccard index (JA). The formulae for each evaluation metric are as follows.

(1) $$AC=\frac{TP+TN}{TP+FP+TN+FN}$$

(2) $$DI=\frac{2\cdot TP}{2\cdot TP+FP+FN}$$

(3) $$JA=\frac{TP}{TP+FP+FN}$$

(4) $$SE=\frac{TP}{TP+FN}$$

(5) $$SP=\frac{TN}{FP+TN}$$

where TP, TN, FP, and FN refer to the number of true positives, true negatives, false positives, and false negatives, respectively.

For diverse tasks, the relative importance of these evaluation metrics varies significantly, depending heavily on the specific application context and requirements of the task. In the domain of segmentation tasks, three principal evaluation metrics emerge as particularly salient, each serving unique roles and possessing varying degrees of relevance:

JA: Also recognized as the intersection-over-union (IoU), JA assesses the degree of overlap between model-generated segmentation outcomes and the actual segmentation results in image segmentation tasks. Consequently, it assumes paramount importance in scenarios necessitating high-precision segmentation.

AC: In image segmentation tasks, achieving precise segmentation of all target regions represents a fundamental requirement. Hence, pixel-level accuracy is the second-most critical metric, ensuring that all target regions are accurately delineated.

SE: Given the typically diminutive size of skin lesions in medical images, segmentation models must exhibit heightened sensitivity to accurately detect these minute skin lesion areas. Therefore, SE takes precedence in this context, enabling the correct identification of all such areas.

In the domain of classification tasks, AC serves as the most fundamental and widely used metric. AC gauges the proportion of correctly classified samples relative to the total sample count, meriting its top rank. Subsequently, SE and SP come into play as crucial metrics to assess the classification efficiency of the model, thus following AC in importance. The JA and DI metrics are principally employed to measure the segmentation efficacy of classifiers. They gain prominence in tasks demanding precise segmentation and are therefore placed fourth and fifth in the order of significance.

### 4.2. Analysis of results

Within the field of image segmentation, DL methods are currently used for most skin diseases to segment skin lesions as a means of identifying diseases and achieving good results [123,124,125]. Table 2 provides a comparative analysis of segmentation algorithms regarding five fundamental evaluation metrics: AC, SE, SP, JA, and DI. Remarkably, there is minimal disparity between ML and DL methods in terms of the AC, SE, and SP metrics. In the context of segmentation tasks, the proper selection and design of features play an indispensable role. Traditional ML techniques often require manual feature design and selection, a process that usually demands specialist expertise. While this method can be laborious and time-consuming, it allows for detailed feature selection. In contrast, DL methods offer the advantage of automatically deriving feature representations from data, reducing the reliance on manual feature engineering. However, it is essential to acknowledge that the quality of this feature selection may not match the meticulous nature of manual feature design. Nevertheless, despite the trade-off in terms of time and human resources, DL methods are still at the forefront in terms of performance. Regarding the JA metric, traditional ML algorithms exhibit a gradual increase in their Jaccard values with enhancements and adjustments in their segmentation methods. However, even with these improvements, the highest threshold typically reached is only 0.81. Notably, this figure falls below the average Jaccard value achieved via DL methods (0.821). This observation highlights the substantial potential of DL algorithms in the domain of image segmentation, surpassing the capabilities of ML techniques. It is particularly noteworthy that the automated utilization of DL methods for segmenting prevalent skin diseases, such as melanoma, has led to significant advancements in this field, providing valuable insights for researchers involved in medical image segmentation [126]. The majority of DL techniques employed in the field of computer imaging predominantly rely on the architecture of CNNs. CNNs are renowned for their characteristics, including local awareness and parameter sharing, and have emerged as the preferred architectural choice for processing image data. They have established themselves as distinguished tools in various applications related to image processing.

In the field of image classification, skin diseases can be systematically categorized into three distinct classes based on their underlying characteristics and clinical manifestations: neoplastic, inflammatory, and pigmented. Table 3 offers a comparative analysis of classification algorithms, with a focus on three key evaluation metrics: AC, SE, and SP (the primary criterion for ranking works from the literature is the methodology used, whether it is ML or DL.). It is crucial to emphasize that the notable variance in accuracy across diverse methods can be ascribed to a multitude of factors. These factors encompass the nature of the classification task, the type of data employed, the specific domain of application, and the scale of the dataset. Classification tasks can be categorized as either multi-class tasks (e.g., four-class or six-class classification) or binary classification tasks. Moreover, the diversity of data types is a pivotal aspect, with clinical images, dermoscopic images, wood images, and others contributing to the variance in classification results. The nature of the region under consideration can also wield a substantial influence on the classification outcomes, whether it pertains to facial areas, complex areas of skin, or smooth areas of skin. Additionally, the size of the dataset plays a pivotal role, with certain tasks involving extensive datasets comprising thousands of images, while others may have smaller datasets with only a few hundred images. Variations in these factors can significantly influence the performance of classification models. Hence, when evaluating disparities between the performances of DL and ML methods, along with their internal mechanisms, it is important to consider their overall trends and levels of accuracy. In the context of neoplastic and inflammatory skin diseases [86,87,88,89,90,91,92,93,94,95,96,97,98,99,100,101,102,103,104], the DL approach has demonstrated a higher classification stability, overall accuracy, and overall specificity relative to the ML method. Particularly noteworthy is the classification accuracy achieved with the BPNN, which reached an impressive 99.7%. This milestone underscores the substantial progress made by DL algorithms in the field of dermatological classification, especially in the realm of neoplastic and inflammatory skin diseases, for which these algorithms have reached a reputable level of application [127]. For pigmented skin diseases, including vitiligo [112,113,114,115], extant classification algorithms are capable of achieving diagnostic accuracies exceeding 85%. This accomplishment is undeniably encouraging. However, the insufficient number of studies in this area leads to these findings having a lack of scientific validity and persuasiveness. Also, the generalization of these models remains inadequately validated, necessitating further scrutiny. While these algorithms may perform well on specific datasets, robust efforts toward validating their broader applicability and accuracy across diverse datasets are required. Addressing these issues may provide substantial prospects for future research endeavors within the field of pigmented skin diseases.

## 5. Discussions

In recent years, researchers have become increasingly interested in ML- and DL-based methods for dermatological diagnosis and have made significant progress [117,128]. Although both ML and DL have achieved very impressive results in the above literature, with the increasing complexity of this task and the increasing amount of data, DL is gradually becoming the dominant method and possesses more potential in this field. Across the published literature, DL methods have shown achievements in dermatological diagnosis comparable to those of dermatologists. A great deal of research and innovative system development is required to develop and validate superior algorithms or systems that support new imaging techniques [129]. Many automated dermatological diagnostic methods have been developed, but a complete decision support system has not yet been fully realized. In this section, we first describe the current state of research. Then, we discuss the main challenges faced by DL in the field of dermatological diagnosis. Instead of describing specific cases in detail, we focus more on the underlying challenges and explain the root causes that lead to these problems. Finally, we try to provide some suggestions for solving these problems.

### 5.1. Current State of Research

This section offers a comprehensive overview of ML- and DL-based algorithms for skin lesion segmentation and classification. In terms of segmentation, substantial progress has been made in the field since its inception in 2015 [130]. The volume of research papers published on skin lesion segmentation published within the last eight years (2015–2022) surpasses the number of papers published in the preceding seventeen years (1998–2014) [131]. However, despite extensive research efforts, skin lesion segmentation remains an unresolved challenge, as evidenced by the ISIC 2018 Skin Lesion Segmentation Field Rankings. While some studies have achieved high Jaccard values, such as reaching 0.94, this does not conclusively establish that DL algorithms have fully conquered the realm of image segmentation. The generalizability and robustness of these methods are yet to be definitively demonstrated, primarily due to constraints related to dataset size and characteristics. Consequently, we posit that the field of skin lesion segmentation via DL warrants further dedicated research.

In terms of classification, there has been remarkable progress in enhancing the diagnostic accuracy and efficiency of dermatological classifications. In past clinical diagnostic scenarios, the accuracy of diagnosis often hinged on the image quality and the experience of the dermatologist, making it highly subjective and susceptible to misdiagnosis. The advent of ML and DL algorithms has led to the development of CAD systems that significantly support dermatologists in diagnosing skin diseases. Evidently, DL-based methods effectively address the limitations of traditional ML-based methods. When compared to traditional ML, DL demonstrates the superior performance, boasting an average classification accuracy of 89.5% and even reaching a maximum of 99.7%. These data affirm the relative development of DL research in the domain of dermatological disease classification. However, unlike segmentation, classification faces a major challenge known as the “black box” problem. In the field of medical image processing, the interpretability of classification results using DL methods remains a major challenge [132].

To better visualize the current state of research on skin lesion detection, a search of the Google Scholar database was conducted on 9 November 2023. Research papers with the keywords “skin lesion classification”, “skin lesion segmentation”, “skin lesion detection” and “skin lesion identification” in their title were screened. The results of this search are shown in Table 4, which shows that the number of publications on skin disease diagnosis is increasing every year. Melanoma and skin cancer were identified as the main areas of interest for skin lesion identification and were discussed in more than 70% of the papers. Among the collected publications, the use of ML and DL in skin disease identification was discussed in 197 papers. Of these papers, 167 mentioned the DL method, while the other 30 used the ML method. Thus, researchers preferred DL methods. The CNN method was reported as the current most popular DL method [133], which is perfectly fitting with its success in image recognition tasks. Besides this, a large number of studies on transfer learning methods have emerged in publications of the last three years [134], which shows the current trend of research in the field of skin image diagnosis.

### 5.2. Challenges

As DL has evolved over the past few years, various efforts to utilize DL methods for dermatological diagnosis have been proposed, with promising performances. However, there are still several issues that need to be addressed before DL can be widely applied to real-world clinical scenarios for use in dermatological diagnoses.

#### 5.2.1. Limitations of Datasets

Limitations in terms of datasets have two main components: the size of the dataset and the type of dataset. Previous work using DL for dermatological diagnoses has typically been trained and tested using datasets with a limited number of images. The largest publicly available dermatology dataset is the ISIC dataset [13], which contains over 20,000 images of skin. While one can obtain any large amount of dermatological data without any diagnostic information from the Internet, labeling such a large amount of dermatological data with diagnoses requires expertise. Nevertheless, training a deep neural network requires a large amount of data for labeling. Too few data can lead to problems such as overfitting. Therefore, larger datasets with diagnostic information are needed to train deep neural networks for effective skin disease diagnosis. In addition to this, imbalances in the samples of the dataset can greatly affect the diagnostic results. For some rare diseases and minority groups, only a limited number of images are available for use in training. A large number of algorithms to date have shown a bias toward such minorities [135], which can lead to a larger gap between their sensitivity and specificity in multi-classification tasks.

#### 5.2.2. Explainability of Deep Learning Methods

DL models are often considered “black box” models. The principles of DL models as “black box” models have not been explained at this stage, which may lead to unpredictable system outputs. Such an “end-to-end” decision-making model results in DL having a weak explanatory power. The internal logic of DL is not clear, making the diagnostic results of this model less convincing. Humans cannot really understand how a machine works, even if it is actually inspired by humans [136]. In the field of medicine, especially for disease diagnosis, interpretable studies of dermatological classification can ensure the reliability and safety of these systems by providing a clear understanding of the behavior and boundaries of the system toward all of its components. Doctors and patients should understand the rationale behind disease diagnosis in order to make informed decisions. Therefore, before DL algorithms are applied in clinical diagnoses, the transparency of the model must be improved.

#### 5.2.3. Homogenized Research Directions

Currently, the majority of studies in both the classification and segmentation domains focus on tumors and inflammatory skin diseases because of their prevalence and associated health risks, particularly melanoma and skin cancer. The prominence of these diseases may stem from their unique features that make them easier to recognize and classify via images. Although, pigmented skin diseases are of equal significance as they also have substantial health implications for patients [129], such as vitiligo. An in-depth study of pigmented skin diseases is equally demanding [137], as they often require in-depth analyses and diagnoses. Moreover, most of the current research has been focused on dermoscopic images due to the fact that dermoscopic images are more often collected and have better properties for segmentation and classification. However, with the increasing use of smartphones, clinical images can be obtained through digital cameras or smartphones easily, which can be valuable in assessing the severity of a patient’s lesions [138]. In order to advance the development and application of clinical images in skin diagnosis, the standard of images captured using smartphones should be consistent with those taken by clinicians during the examination of skin lesions, and the relevant features of these images should fully support the diagnosis.

#### 5.2.4. More Innovative Algorithms Are Needed

After summarizing and comparing the current algorithms, it was found that there are some key limitations that make it difficult for the reported algorithms to achieve significant improvements in their diagnostic accuracy. As shown in the examples from the literature presented in the previous sections, most existing dermatological diagnostic tasks usually use the current popular DL architectures for image segmentation or classification, and the ML architectures have not changed much; secondly, the ML architectures for different types of dermatological diseases are uniform, and very few studies select the appropriate type of deep neural network for the specific dermatological diagnostic task [139]. Finally, the current DL algorithms mainly rely on unimodal data as inputs in many tasks, which leads to overly homogeneous and insufficiently comprehensive criteria for classification and diagnosis. In the case of unimodal data, algorithms can only make decisions based on information from that modality, lacking the richness of information available from multimodal data.

### 5.3. Future Directions

AI has made great strides in the field of dermatological recognition, and the use of DL methods in dermatological diagnosis is becoming increasingly popular. Nevertheless, as discussed above, there are still some challenges in this field that need to be actively addressed in order to achieve satisfactory diagnostic results. In order to address these challenges and achieve better dermatological diagnosis results, future research and practices can explore and focus more effort on the following areas.

#### 5.3.1. Establish a Standardized Dermatological Image Dataset

To facilitate the training of more precise and resilient deep neural networks, it is imperative to assemble larger, more diverse, and more representative skin lesion image datasets that encompass manually labeled segmentation outcomes for each image. Automated or semi-automated data labeling tools, such as Fiji, LabelMe, and Imagetagger, can be used to efficiently label massive amounts of data [140]. In addition, there are some problems with the existing publicly available skin image datasets. Current public datasets such as the ISIC contain dermatological images of light-skinned subjects primarily from the United States, Europe, and Australia. While current disease recognition models perform well in these subjects, their efficacy has been questioned when dealing with dermatological images of individuals from other geographic regions [141]. Therefore, there is a need for more balanced and standardized datasets that include clinical data from different regions, genders, ages, skin types, and ethnicities in order to improve the generalization performance of the models.

#### 5.3.2. Provide Reasonable Explanations for Predicted Results

Interpretability is an important factor that limits the application of DL methods in clinical diagnostic scenarios. In order to improve the interpretability, visualization tools and techniques can be used, which can help users to understand the decision-making process of the model. For example, by using heatmaps, the importance of features can be visualized, while by visualizing decision trees, the branching structure of the tree model can be clearly presented. Therefore, the research and development of interpretable tools and techniques is a key direction for the field of DL, contributing to the transparency of models and the trust of users. In addition, one possible solution is to start from the aspect of feature representation, providing reasonable explanations for predictions based on the ABCDE criteria or the seven-point skin lesion malignancy checklist [142]. Interpretability also has the potential to monitor ethical and compliance-related issues raised by biases in training data. It provides a more effective mechanism to address the bias and auditing issues posed by AI [143].

#### 5.3.3. Increase the Diversity of the Types of Research

Researchers need to refocus their attention beyond just concentrating on areas concerning melanoma and skin cancer. While the emphasis on melanoma is undoubtedly critical for life-threatening reasons, we must recognize that the broader spectrum of skin conditions that dermatologists face in their clinical practice, including a wide range of benign lesions, is equally important. Future research efforts should aim to achieve a more comprehensive classification and segmentation of dermatological conditions, including neoplastic, inflammatory, and pigmented skin diseases, in order to establish a more comprehensive basis for medical diagnoses and treatment. This will require encouraging researchers to develop systems that can accurately segment and classify different types of skin lesions. In addition, future research should focus more on clinical images rather than dermoscopic images to better meet the practical needs of dermatologists.

#### 5.3.4. Actively Explore Innovative Models and Methods 

Changing technological routes and optimizing algorithms is the most focal way to overcome the current challenges. Innovations in algorithms and technology should be considered in the following areas:

(1) Try to use the latest model architecture. The Swin transformer is a new model of computer vision proposed in 2021 with a wide applicability for tasks including image segmentation, image recovery, and image reconstruction [144,145]. Currently, only a limited number of studies have reported the use of this model for medical images [146,147,148]. The Swin transformer, through its hierarchical structure and self-attentive mechanism, has shown powerful feature extraction and modeling capabilities in medical image processing, providing potential opportunities for improvement and innovation in medical image analysis and diagnosis [149]. This suggests that the application of modified models in medicine deserves further research.
(2) Innovate areas of utilization of traditional methods. As mentioned above, there have been many works utilizing transfer learning techniques to improve the performance of DL models in dermatological diagnosis tasks [105,106,107]. At the same time, recent developments regarding transfer learning in other domains can be utilized to facilitate the success of DL in dermatological diagnosis tasks. It is worth mentioning that Reinforcement Learning (RL) also has the potential to be applied to dermatological diagnosis. Recent studies have demonstrated the successful application of RL in different domains [150], partly due to the powerful function approximation capabilities of DL algorithms. RL is very effective in dealing with sequential problems, and many medical decision-making problems fall into this category. Therefore, RL can be used to solve these problems. There have been some works utilizing RL to solve medical image processing tasks with promising results [151]. However, there is no work that applies RL to dermatological diagnostic tasks. Thus, RL may be a potential tool for solving the problems regarding dermatological diagnosis.
(3) Fusion of multimodal data. Integrating multi-source data such as images, text, and biological features can enhance the performance of dermatological diagnostic models. In a clinical setting, accurate dermatological diagnosis is not only dependent on a single skin image, but also requires the consideration of additional clinical information such as medical history, risk factors, and overall skin assessment. Some studies have verified significant improvements in diagnostic performance including these additional data and close-up images [118]. Therefore, this information could be incorporated into the process of model training and testing for skin disease diagnoses. In addition, medical record data can also be processed using techniques such as document analysis [152] and data mining [153], and taken into account in the diagnostic process as well. This multimodal data fusion model has proven its effectiveness in recent studies and can be extended to the field of dermatological diagnosis.

## 6. Conclusions

The development of AI-based diagnoses of skin lesions is a research area of great interest, which benefits from appropriate methodologies and abundant datasets that are continuously updated. Despite the progress made in the last decade, many aspects still require further investigation and improvement.

In this paper, we review methods for the segmentation and classification of skin lesions using ML and DL, and discuss the current state of research, its challenges, and future directions for DL-driven dermatological diagnosis. These studies help to enhance the development of advanced concepts and methods. In summary, there is a need for establishing more standard datasets, developing more visualization tools, innovating model architectures, and fusing multiple techniques in the future to comply with the trends of dermatological image segmentation and classification. There is a particular need to develop more reliable automated diagnostic methods when faced with an increasing variety of clinical data.

## Figures and Tables

**Table 1:** Three types of imaging equipment, their imaging standards, and their applicable models [20,21,22,23,24,25,26,27,28,29,30].

Imaging Equipment	Skin Imaging Standards	Model of Applicability
**Dermoscopy**	1. Overall skin lesion: natural light was used as the light source, and the mode and magnification were noted. 2. Local details: the maximum magnification and clear images of the skin lesions were taken.	AlexNet, VGG, GoogLeNet, ResNet, CNN [20,21,22,23,24].
**RCM**	1. Longitudinal scanning: we scanned from the stratum corneum to the superficial dermis; each layer’s thickness was 5 μm. 2. Horizontal scanning: pathological changes in the stratum corneum, stratum granulosum, stratum spinosum, stratum basale, dermo-epidermal junction, and superficial dermis were scanned. 3. Local details: for each layer of pathological changes, photos of local details were taken.	SVM, CNN, InceptionV3, Bayesian model, Nested U-net [25,26,27,28,29].
**VHF skin ultrasound**	1. Longitudinal scanning: the lesion area was scanned using high-frequency or ultra-high-frequency ultrasound, and the scanning frequency (20 MHz, 50 MHz, etc.) was marked. 2. Overall and detailed imaging: it was able to clearly display the epidermis, dermis, and subcutaneous tissue, and measure the range, depth, blood flow, and nature of skin lesions and their relationship with surrounding tissues.	DenseNet-201, GoogleNet, Inception-ResNet-v2, ResNet-101, MobileNet [30]

**Table 2:** Performance comparison of various dermatological segmentation algorithms.

Reference	Method	AC	SE	SP	JA	DI
[51]	LSC (ML, 2015)	96.2%	92.6%	-	0.81	-
[52]	K-means (ML, 2016)	90%	-	-	-	-
[53]	RGB threshold (ML, 2019)	-	-	-	0.789	0.876
[53]	XYZ threshold (ML, 2019)	-	-	-	0.8	0.884
[54]	ICA (ML, 2019)	-	99.49%	98.46%	0.7087	-
[56]	FCM (ML, 2020)	90.89%	92.84%	88.27%	-	-
[58]	FCN (DL, 2017)	95.3%	93.8%	95.2%	0.841	0.907
[60]	FCDN (DL, 2017)	99.53%	87.9%	97.9%	0.783	0.865
[61]	DFCN (DL, 2017)	93.4%	82.5%	97.5%	0.765	0.849
[62]	FCRN (DL, 2017)	85.5%	54.7%	93.1%	-	-
[63]	SegNet (DL, 2021)	-	95.6%	95.42%	-	0.749
[63]	U-Net (DL, 2021)	-	96.4%	94.8%	-	0.733
[64]	FCN (DL, 2018)	-	-	-	0.884	-
[65]	ResNet34 (DL, 2019)	-	-	-	0.768	0.851
[66]	U-Net (DL, 2019)	97%	90%	99%	0.88	0.94
[67]	FCN U-Net (DL, 2019)	90%	96%	-	0.83	-
[68]	U-Net, VGG-16 (DL, 2021)	96.7%	90.4%	98%	0.846	0.915
[69]	MSFCDN (DL, 2018)	95.3%	90.1%	96.7%	0.785	0.869
[70]	DPFCN (DL, 2019)	98.9%	92.4%	99.6%	0.852	0.916
[71]	ResU-NeXt ++ (DL, 2021)	96%	-	-	0.8684	0.9235
[72]	U-Net (DL, 2022)	90.74%	-	-	0.7572	-
[73]	DeepLabv3 + (DL, 2023)	95%	90%	90%	-	-
[75]	W-EFO-E-CNN (DL, 2023)	98%	99.54%	50%		0.987
[76]	DCNN (DL, 2019)	-	-	-	0.714	-
[77]	U-Net (DL, 2020)	-	-	-	0.887	-
[78]	SEDSIC (DL, 2021)	97%	98%	96%	0.94	0.97
[79]	FCN-UTA (DL, 2021)	-	86.36%	-	0.7381	0.8493

**Table 3:** Comparison of classification algorithms for various skin diseases.

Reference	Methods	AC	SE	SP	Classes	Data Type	Data Size
[86]	Adaboost (ML, 2015)	89.35%	93.5%	85.2%	2	Dermoscopic images	-
[87]	KNN–SVM (ML, 2015)	85%	-	-	5	Clinical images	726
[88]	SVM (ML, 2016)	96.8%	95.4%	89.3%	2	Dermoscopic images	320
[89]	KNN–SVM (ML, 2017)	90%	-	-	4	Dermoscopic images	-
[90]	SVM (ML, 2018)	92.3%	-	-	3	Dermoscopic images	-
[91]	SVM (ML, 2019)	89.43%	91.15%	87.71%	2	Dermoscopic images	1000
[92]	Naive Bayes (ML, 2020)	72.7%	91.7%	70.1%	6	Dermoscopic images	1646
[93]	CNN (DL, 2020)	75%	73%	78%	2	Dermoscopic images	1796
[94]	BLSTM (DL, 2022)	89.47%	88.33%	97.17%	7	Dermoscopic images	10,015
[95]	Eff2Net (DL, 2022)	84.70%	84.70%	-	4	Clinical images	17,327
[96]	BPNN (DL, 2020)	99.7%	99.4%	100%%	2	Dermoscopic images	400
[97]	DenseNet201 (DL, 2022)	95.5%	93.96%	97.06%	2	Dermoscopic images	3297
[98]	Inception-ResNet-V2 (DL, 2020)	87.42%	97.04%	96.48%	4	Clinical images	14,000
[99]	Inception-ResNet V2 (DL, 2019)	89.63%	77%	-	6	Clinical images	11,445
[100]	GoogleNet (DL, 2020)	99.29%	99.22%	99.38%	2	Dermoscopic images	2376
[101]	AlexNet (DL, 2020)	98.7%	95.6%	99.27%	7	Dermoscopic images	10,015
[102]	GoogleNet (DL, 2020)	94.92%	79.8%	97%	8	Dermoscopic images	29,439
[103]	RDCNN (DL, 2022)	97%	94%	98%	2	Dermoscopic images	2206
[104]	InSiNet (DL, 2022)	94.59%	97.5%	91.18%	2	Dermoscopic images	1471
[105]	GoogleNet(Inception-V3) (DL, 2020)	83.78%	87.5%	79.41%	2	Dermoscopic images	1471
[106]	DenseNet-201 (DL, 2020)	87.84%	95%	79.41%	2	Dermoscopic images	1471
[107]	ResNet152V2 (DL, 2020)	86.49%	92.5%	79.41%	2	Dermoscopic images	1471
[108]	DenseNet, ResNet (DL, 2023)	95.1%	92%	98.8%	7	Dermoscopic images	10,015
[109]	U-Net, CNN (DL, 2023)	97.96%	84.86%	97.93%	7	Dermoscopic images	10,015
[110]	DCNN (DL, 2023)	97.204%	97%	-	7	Dermoscopic images	10,015
[111]	Visual Transformer (DL, 2023)	93.81%	90.14%	98.36%	7	Dermoscopic images	10,015
[112]	Resnet50, VGG16, Inception v2 (DL, 2019)	87.8%	90.9%	91.9%	2	Clinical images	38,677
[113]	Cycle GAN, ADRD, Resnet50 (DL, 2020)	85.69%	-	90.92%	2	Wood lamp images	10,000
[114]	YOLO v3, PSPNet, UNet ++ (DL, 2022)	85.02%	92.91%	-	3	Clinical images	2720
[115]	LVQ Neural Network (DL, 2017)	92.22%	-	-	3	Clinical images	1002

**Table 4:** Number of publications relating to skin diagnosis.

Year	2018	2019	2020	2021	2022	2023
Publication No.	104	127	161	190	245	233
